# Modern Medico-Psychology and Psychiatry

**Published:** 1895-05-11

**Authors:** T. S. Clouston

**Affiliations:** Lecturer on Mental Diseases, Edinburgh University, and Physician Superintendent Royal Asylum, Morningside


### Modern Medico-Psychology and Psychiatry.

By T. S. Clouston, M.D., Lecturer on Mental Diseases, Edinburgh University, and Physician Superintendent Royal Asylum, Morningside.

### THE CLINICAL CLASSIFICATION OF THE INSANITIES.

The various forms of mental disease which I have briefly described in the previous papers are chiefly segregated from each other by mental differences. The principle of their differentiation was psychological and not somatic. The classification was, in short, essentially a mental one. Such a classification is necessarily one of symptoms, not of pathological changes in the brain structure. It is somewhat as if we classified chest diseases by the coughs which are their most distressing symptoms, or nervous diseases by the kinds of pain they cause. Not that this analogy goes far, for mind so infinitely transcends and dominates all other functions, and consciousness and personality are so evidently the "greatest things in man," that any real classification of morbid mental symptoms must have interest and authority without reference to the brain or body at all. The weak points of this mental classification are: (1) That there are many instances where the symptoms change, so that a case is one of mental depression at first, then becomes one of mental exaltation, and ends in incurable mental enfeeblement, so that it is one of melancholia, mania, and dementia at different times; and (2) that we are not sufficiently helped in our diagnosis, prognosis, and treatment of our cases by this system. Many men have, therefore, sought out new and better modes of classifying mental diseases. Every one of these have had more direct reference to brain and body than the Pinel-Esquirol mental classification. The discovery of general paralysis by Bayle, about 1820, made nosological content and finality impossible thereafter. Here was a real disease of the brain, with definite bodily and mental symptoms conjoined, running a definite course, in which all the mental and bodily symptoms might completely change, and after death exhibiting definite pathological changes in the brain. The disease from beginning to end was a real entity; yet in the same case there may be states of mental exaltation, of depression, of enfeeblement, and of stupor, succeeding each other. To name such a true disease "general paralysis," gives a totally different, and a far more complete knowledge of it, than to call it mania or melancholia, as cases of it would have been called before 1820.

Morel, in France, elaborated a classification founded on a combination of bodily causes and symptoms which led the way to the still more useful system of classification, devised by Dr. Skae, my predecessor here, and published by him in 1863. This is now commonly called the clinical classification. It is eminently British in character, not being strictly logical and consistent, or altogether scientific, but yet most practical and helpful to the practitioner of medicine. The best proof of this is that it has in whole, or in part, slowly crept into every good book on mental diseases since published, and its terms have become psychiatric necessities, and this in spite of much opposition and foolish jealousy. As Skae puts it, he founded this classification on the "natural history" of the diseases he had to consider. In a large degree it is founded on the bodily causation of these diseases, and is therefore now commonly called the "Somato-Etiological" classification. Its great merit is that it helps the practising physician in his efforts to discover the causes of the insanity, and also assists him in his treatment and prognosis. No other mode of assorting mental diseases does half as much for us in these respects as Skae's. If I were to describe its principles I would say that it seizes on the bodily and constitutional relationships of the mental symptoms, and groups the latter accordingly. By such grouping it is found that we get similarities of course and of clinical characteristics, and our insanities come therefore to be clinical entities like other bodily diseases. Until we reach that stage of knowledge when we can form a complete pathological classification—of which we now have a few varieties, general paralysis being the chief—I think Skae's grouping by far the most useful yet devised; and even after we have got a perfect classification, we shall have to take very careful account, as practitioners, of Skae's groups. In spite of the favour with which Krafft-Ebing's more recent classification is regarded in some quarters, I adhere strongly to this opinion.

The nature and practical usefulness of Skae's clinical classification will be best realised by taking a few illustrations. He took the various physiological periods of life, and finding that each was liable to be accompanied by a variety of mental defects or disturbances peculiar to itself, and each tinctured by the bodily and mental characteristics of its period, he grouped the insanities of the "times of life" into "congenital," "pubescent and adolescent," "climacteric" and "senile" insanities. Each one of these has, in a considerable degree, its own special mental characters, its own bodily symptoms and accompaniments, and its own course and terminations. A doctor knows far more about the symptoms, the course, and the right treatment of any case if he is told it is one of adolescent insanity than if it is said to be one of mania or paranoia. Then Skae grouped all the cases that had relationship to reproduction into the "hysterical," "masturbational," "uterine and ovarian," "post-connubial," "pregnancy," "puerperal," and "lactational" insanities. No such vivid light can be cast on any case of mental disease by any one word as to say it is a "puerperal" one. It spells urgency, acuteness, risk, amenability to treatment, and a favourable issue, all combined, in a way no other word does. Then the "alcoholic," "rheumatic," "epileptic," and "phthisical" insanities each throw light, not only on the causes of each case and on its symptoms, bodily and mental, but above all on its appropriate modes of treatment and on the issue to be reasonably expected. It is quite true that the special mental aspects and symptoms of many of these somato-etiological varieties are not always distinguishable at once, and by one examination of the case. One needs to inquire into the causation and the history of the case, and its bodily as well as its mental symptoms, and not at one period only, but in some instances for a time, to be able to classify it. But this difficulty merely shows how closely this classification follows the whole of the clinical facts of disease. It is now common to combine the mental and the clinical classifications by using such terms as "adolescent mania," "climacteric melancholia," and "senile dementia." The mental classification marks the genus and the clinical the species. Thus we get the benefit of both.

